# Comprehensive Review on DNA Hydrogels and DNA Origami-Enabled Wearable and Implantable Biosensors

**DOI:** 10.3390/bios15120819

**Published:** 2025-12-18

**Authors:** Man Li, Joonho Bae

**Affiliations:** Department of Physics and Semiconductor Science, Gachon University, Seongnam-si 13120, Gyeonggi-do, Republic of Korea

**Keywords:** DNA nanomaterial, DNA hydrogel, DNA origami, wearable biosensor, implantable biosensor

## Abstract

DNA nanoparticles have emerged as potent platforms for wearable and implantable biosensors owing to their molecular programmability, biocompatibility, and structural precision. This study delineates two principal categories of DNA-based sensing materials, DNA hydrogels and DNA origami, and encapsulates their fabrication methodologies, sensing mechanisms, and applications at the device level. DNA hydrogels serve as pliable, aqueous signal transduction mediums exhibiting stimulus-responsive characteristics, facilitating applications such as sweat-based cytokine detection with limits of detection as low as pg·mL^−1^ and microneedle-integrated hydrogels for femtomolar miRNA sensing. DNA origami offers nanometer-scale spatial precision that improves electrochemical, optical, and plasmonic biosensing, as shown by origami-facilitated luminous nucleic acid detection and ultrasensitive circulating tumor DNA assays with fM-level sensitivity. Emerging integration technologies, such as flexible electronics, microfluidics, and wireless readout, are examined, alongside prospective developments in AI-assisted DNA design and materials produced from synthetic biology. This study offers a thorough and practical viewpoint on the progression of DNA nanotechnology for next-generation wearable and implantable biosensing devices.

## 1. Introduction

In recent years, DNA nanotechnology has evolved from early conceptual motifs into a mature material platform encompassing both large, soft DNA hydrogel [1,2,3] networks and atomically precise DNA origami [4,5] nanostructures. These two classes of materials share inherent advantages, including modularity, chemical definability, and compatibility with aqueous processing, which align well with the core requirements of in vivo and ex vivo biosensing [6,7,8]. In particular, their intrinsic modularity allows the independent design and regulation of network architecture, crosslinking chemistry, and functional groups, thereby enabling the optimization of mechanical properties, molecular transport efficiency, and molecular recognition performance in biosensing applications. Compared with other nanomaterials, DNA offers exceptional chemical purity and highly precise structural geometry [9,10]. As DNA is composed of nucleotides with a uniform and impurity-free chemical composition and is readily modifiable, DNA hydrogels can form soft, water-rich three-dimensional (3D) networks that mimic the mechanical characteristics of human tissues, making them ideal for skin-adherent or -implantable flexible sensing devices [11,12]. Furthermore, the high structural precision and predictability of DNA enable DNA origami to position captured probes and redox labels on electrode surfaces with nanometer accuracy, thereby precisely modulating interfacial electron transfer kinetics [13,14]. Collectively, these attributes allow DNA-based materials to address two central demands of biosensing: (i) maintaining a hydrated, stable, and comfortable interface with skin or tissues (achieved through DNA hydrogels for patches, microneedles, and wearable devices) and (ii) achieving nanoscale spatial control on electrode surfaces to ensure high signal fidelity and reliability (achieved through DNA origami for electrochemical sensing) [15,16,17]. Thus, integrating DNA hydrogels or DNA origami with biosensing technologies enables continuous, minimally invasive molecular monitoring under safe and comfortable conditions.

In this review, DNA-based sensing materials are categorized into two major groups, and recent advances in DNA hydrogels and DNA origami nanotechnologies are systematically summarized. In wearable biosensing applications, both pure DNA hydrogels and hybrid DNA hydrogel systems exhibit outstanding stimuli-responsive behavior, undergoing structural or functional changes upon exposure to specific molecules, thereby enabling highly sensitive detection of target analytes. These features make them particularly well suited for the continuous monitoring of biomarkers in sweat and other body fluids. Meanwhile, DNA origami offers exceptional structural programmability and recognition precision. Through the programmable self-assembly of DNA, a wide range of two- and three-dimensional (2D or 3D) nanostructures can be constructed, providing highly controllable molecular platforms capable of accurately recognizing proteins, nucleic acids, viruses, and other biomolecules. For clarity, this review offers a balanced overview of DNA hydrogels and DNA origami. It mainly highlights DNA hydrogels used in wearable and skin-interfaced sensing, while DNA origami is explored concerning exact nanoscale biosensing applications.

## 2. DNA Hydrogels as Adaptive Transduction Media

### 2.1. DNA Hydrogels Fabrication Strategies and Structural Features

#### 2.1.1. Pure DNA Hydrogels

**Figure 1:**
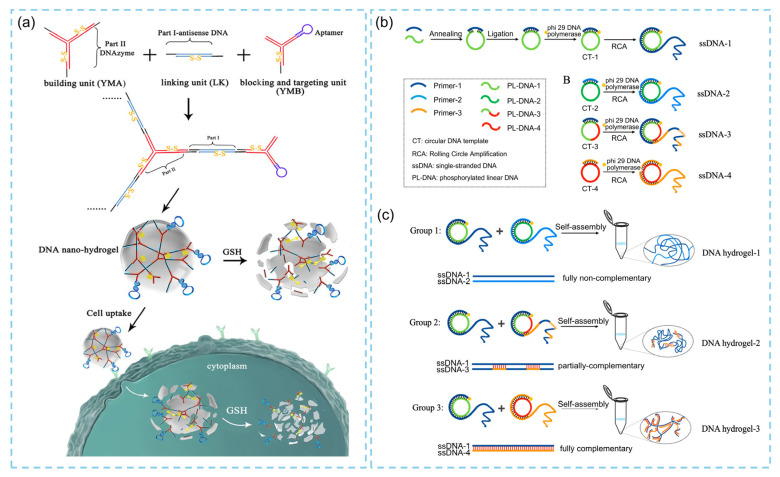
Examples of pure DNA hydrogels. (**a**) Schematic illustration of DNA hydrogel formed with “sticky end” segments [20]. (**b**) Double rolling circle amplification (RCA) method for preparing four circular ssDNA templates. (**c**) DNA hydrogels formed by ssDNA self-assembly [23].

Pure DNA hydrogels were first reported in 2006 [18]. These materials are typically assembled from short, branched oligonucleotide motifs, such as X-, Y-, or T-shaped DNA. The motifs connect through complementary single-stranded “sticky ends” to form a three-dimensional crosslinked network. By programming the sequences of the sticky ends and exploiting Watson–Crick base pairing, the spacing between crosslinks and the pore size of the network can be precisely controlled at the nanoscale [1,19]. In these systems, DNA monomers act simultaneously as both crosslinkers and structural building units. Adjusting the concentration and type of the starting DNA monomers enables tuning of the hydrogel’s mechanical and functional properties for diverse applications. For example, in 2015, Li et al. [20] constructed a functional DNA hydrogel capable of recognizing specific cancer cells using Y-shaped DNA. The hydrogel was assembled by hybridizing the sticky ends of Y-shaped monomer A (YMA) and monomer B (YMB) with complementary segments on a DNA linker (LK), and additional functional modules were subsequently incorporated to impart stimulus-responsive behaviors (Figure 1a). However, such approaches rely on high concentrations of DNA for self-assembly, which may introduce cumulative structural errors and lead to increased costs [21]. To address these limitations, researchers have developed nucleic-acid-amplification-based strategies for constructing pure DNA hydrogels, including hybridization chain reaction (HCR) [22], rolling circle amplification (RCA), and multi-primer chain reaction (MCA). Among these, RCA-derived networks have attracted particular attention due to their mild, isothermal reaction conditions. During RCA, a circular template and a strand-displacing polymerase generate ultra-long single-stranded DNA (ssDNA) concatemers that entangle with one another and, when the template encodes repeating binding domains, crosslink in situ to form bulk hydrogels. In 2023, researchers used dual-RCA to produce four ssDNA precursors with different degrees of hydrogen bonding (Figure 1b) [23]. The hydrogen-bonding strength between ssDNA chains adjusted by turning the circular template sequences, thereby enabling efficient fabrication of large-scale DNA hydrogels with adjustable mechanical properties (Figure 1c). Moreover, RCA-derived networks can be encoded with functional repeating sequences, such as sticky ends, i-motifs, or G-rich segments, providing the hydrogels with intrinsic signal-generation capabilities [12]. One representative example is the incorporation of G-quadruplex (G4)–heme complexes as catalytic motifs. G-rich sequences fold into G-quadruplex structures and coordinate with heme to form stable peroxidase-mimicking catalysts, capable of converting H_2_O_2_ into colorimetric or electrochemical signals using common substrates (e.g., TMB, ABTS) or mediators. Because the catalytic modules are genetically encoded within the DNA and assembled during gel formation, these systems eliminate the need for fragile protein enzymes and enable robust signal generation using simple reagents at room or physiological temperatures [24].

#### 2.1.2. Hybrid DNA Hydrogels

**Figure 2:**
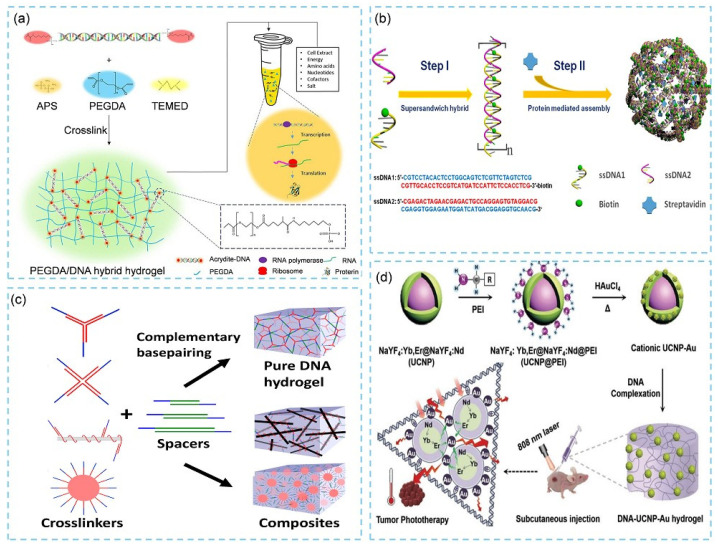
(**a**) The schematic illustration of the preparation of PEGDA/DNA hydrogel for cell-free protein production [30]. (**b**) Programmable self-assembly of DNA−protein hybrid hydrogels [31]. (**c**) Schematic illustrations of DNA-based pure hydrogel and hybrid hydrogel formation [32]. (**d**) Schematic illustration for the fabrication of DNA–inorganic hybrid hydrogels (DNA–UCNP-Au) [33].

Before the concept of pure DNA hydrogels, Nagahara et al. had reported hybrid DNA hydrogels in 1996 [25]. These hydrogels typically consist of two components: a primary backbone material and DNA-based crosslinkers. The backbone material forms the structural framework of the hydrogel and may include synthetic polymers [26] (e.g., polyacrylamide, polyethylene glycol, polypeptides, and proteins), carbon nanotubes (CNT) [27], or graphene oxide (GO) [28,29]. DNA serves as the crosslinking element that bridges the backbone units to generate a three-dimensional (3D) network. In 2020, Cui and colleagues developed a low-cost PEGDA/DNA hybrid hydrogel for cell-free protein synthesis (CFPS) using polyethylene glycol diacrylate (PEGDA) as the backbone (Figure 2a) [30]. This design significantly reduced the amount of DNA required, thereby lowering the overall material cost by more than 30 times. In 2016, researchers employed an ultra-sandwich hybridization strategy to prepare double-stranded DNA (dsDNA) modules containing precisely quantified biotin groups [31]. Streptavidin was then used to trigger the formation of flower-like porous DNA–protein hybrid hydrogels with an average diameter of 6.7 ± 2.1 μm (Figure 2b). These hydrogels exhibited excellent biocompatibility and were suitable for enzyme encapsulation and storage. Beyond chemical crosslinking strategies, DNA can also interact with inorganic materials through physical adsorption to form DNA–inorganic hybrid hydrogels. For instance, in 2021, Gao’s group prepared DNA-coated carbon nanotubes via π–π stacking between ssDNA and CNT surfaces, and DNA-linked gold nanoparticles through electrostatic interactions between adenine and gold. Mixing these crosslinkers with spacer solutions enabled rapid gelation within 1 min (Figure 2c) [32]. In 2020, Liu et al. [33] constructed an injectable, near-infrared (NIR) responsive DNA–inorganic hybrid hydrogel through electrostatic assembly between DNA and upconversion lanthanide–gold hybrid nanoparticles (UCNP–Au NPs). Compared with the original inorganic nanoparticles, the resulting hydrogel exhibited reduced cytotoxicity and a faster temperature-increase rate under the same NIR irradiation (Figure 2d). Overall, hybrid DNA hydrogels integrate DNA with synthetic polymers or nanomaterials to offer tunable mechanical properties, transport behavior, and interfacial interactions. Polymer backbones enhance toughness and elasticity, facilitating processing and skin adhesion. Zwitterionic or PEG-like components reduce nonspecific adsorption and improve antifouling performance. Conductive or plasmonic nanofillers introduce new modes of signal transduction. Because the recognition unit (DNA) and the mechanical scaffold (polymer/nanomaterial) are functionally decoupled, hybrid hydrogels maintain a high degree of modularity and can be readily reprogrammed for sensing applications targeting diverse analytes [11,34].

### 2.2. Stimuli-Responsive Designs of DNA Hydrogels

**Figure 3:**
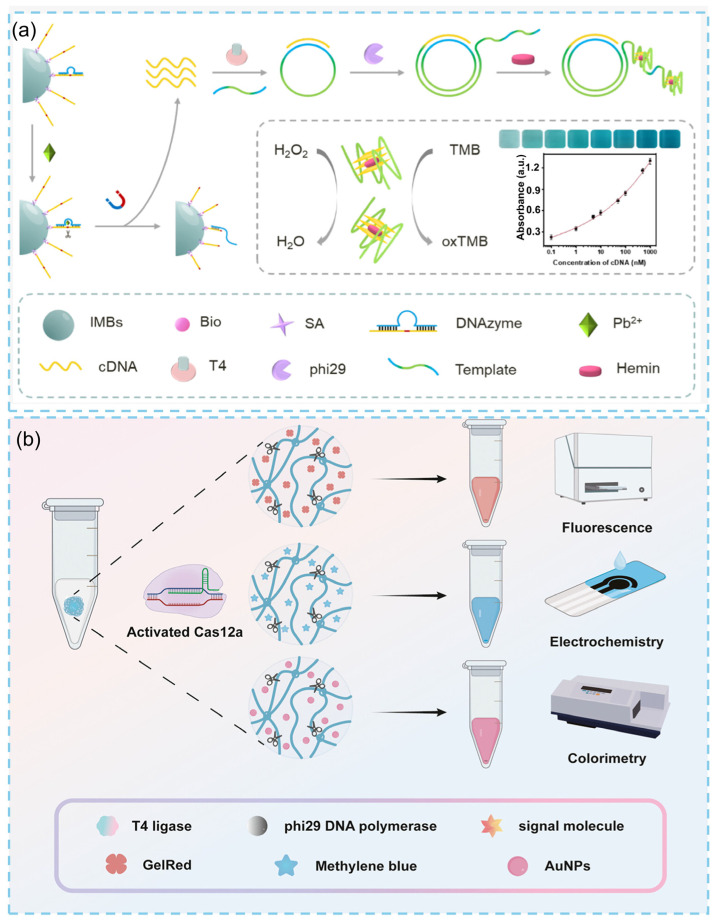
(**a**) Schematic illustration of the Pb^2+^ colorimetric biosensor [43]. (**b**) Nucleic acid detection based on Cas12a trans-cleaves ssDNA regions in the RCA hydrogel [44].

DNA hydrogels can be rationally engineered to respond to a wide range of chemical and biological stimuli, including pH [35,36,37], metal ions [38,39], proteins [40,41], and nucleic acids [42], enabling target-triggered structural or functional transformations. These features make them powerful materials for biosensing applications. For example, incorporating G-quadruplex (G4)–heme DNAzymes into hydrogel matrices imparts enzyme-free catalytic activity. This complex exhibit peroxidase-like properties, has been thoroughly characterized, and is widely employed in biosensing workflows. Such catalytic hydrogels can generate visible color changes, chemiluminescence, or electrochemical signals [6]. In the study by Wang et al. [43], a highly sensitive colorimetric assay for Pb^2+^ detection was developed by integrating a Pb^2+^-specific DNAzyme with a RCA-generated G4/hemin complex. The G4/hemin catalytic system converts the colorless TMB–H_2_O_2_ substrate into a blue oxidized product, thereby markedly amplifying the colorimetric signal and enabling precise quantification of trace Pb^2+^ levels (Figure 3a). By encoding pH-responsive domains (e.g., cytosine-rich i-motif sequences), metal-ion recognition motifs (e.g., T–Hg^2+^–T or G–Ag^+^–C coordination structures), or nucleic-acid anchoring regions into the hydrogel network, these materials can undergo swelling/shrinking, sol–gel transitions, or release of reporter molecules upon target recognition [7]. These structural changes can be detected visually or electronically, forming the basis of numerous self-reporting biosensors or smart drug-delivery systems. For instance, pH-responsive DNA hydrogels can reversibly assemble and disassemble under near-physiological pH, while ATP-aptamer–crosslinked hydrogels liquefy in the presence of intracellular ATP, releasing encapsulated probes or therapeutic agents. In addition, DNA hydrogels can be embedded with fluorophores, redox mediators, or nanoparticle-based reporters, and can be integrated with strand-displacement circuits to achieve isothermal signal amplification, which is an essential feature for low-power wearable and implantable devices. For instance, in 2024, Zhang and colleagues established a CRISPR-responsive biosensing platform based on DNA hydrogels for ultrasensitive nucleic acid detection. This platform supports multiple programmable signal outputs, including fluorescence, electrochemical, and colorimetric readouts, allowing flexible adaptation to diverse detection scenarios and highlighting its broad application potential (Figure 3b) [44].

### 2.3. DNA Hydrogel-Based Wearable Biosensors

**Figure 4:**
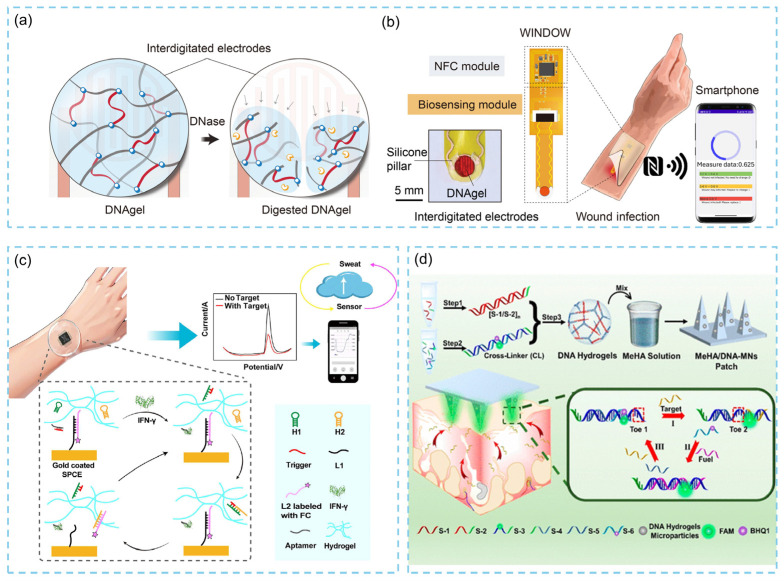
(**a**) Schematic of the infection sensing mechanism. DNAgel is degraded upon exposure to DNase, resulting in a change in the capacitance of the sensor. (**b**) Schematic of the wireless wound infection sensor [45]. (**c**) Schematic diagram of the DNA hydrogel-based wearable electrochemical sensor and the target-triggered reduction in electrical signal [46]. (**d**) Preparation of MeHA/DNA-MNs patches and used for in situ rapid sampling and sensitive detection of microRNA in ISF [49].

Among various biosensing technologies, DNA nanogels have emerged as promising materials for wearable biosensors owing to their high programmability, excellent biocompatibility, structural flexibility, and precise target responsiveness. These features enable their widespread application in health monitoring, iontronic skins, strain sensing, and related fields. For example, in 2021, researchers introduced a flexible wireless infection detection on wounds (WINDOW) for noninvasive monitoring of wound infections [45]. This system employs a customized DNA hydrogel (DNAgel) capable of producing radio-frequency–detectable signals in response to deoxyribonuclease (DNase) (Figure 4a). DNA is chemically crosslinked and gelled in situ at the bioelectronic interface, allowing seamless integration with flexible electronic components. Because DNase associated with bacterial activity selectively degrades DNA hydrogels, this degradation serves as an indicator of infection. When embedded into a thin, compliant wound dressing, the DNAgel undergoes nonspecific strand cleavage and dissolution upon exposure to extracellular DNase, altering the dielectric constant above the interdigitated electrodes. The resulting electrical change is then wirelessly read through near-field communication (NFC) without external power, enabling real-time monitoring of wound infection and facilitating precise postoperative or chronic wound management (Figure 4b). Recently, Dai and colleagues combined functional DNA hydrogels with screen-printed carbon electrodes (SPCEs) to construct a wearable electrochemical biosensor for sweat collection and interferon-γ (IFN-γ) detection [46]. An IFN-γ aptamer was first integrated into the hydrogel and hybridized with a trigger strand (TS). In the presence of IFN-γ, aptamer–target binding releases the TS, which subsequently initiates cyclic hairpin hybridization amplification (CHA) between hairpins H1 and H2. This reaction causes ferrocene probes (L2) on the electrode surface to dissociate, resulting in a decrease in current signal (Figure 4c). Benefiting from the high loading capacity and excellent sweat enrichment properties of the DNA hydrogel, the sensor achieves highly sensitive detection of IFN-γ in sweat. In practical wearable and implantable settings, the biocompatibility and durability of DNA-based materials over time are essential for reliable sensing. DNA hydrogels can degrade due to nuclease activity, ionic crosslink breakdown, and dehydration during prolonged skin contact. However, approaches like backbone modifications (such as phosphonothioate stabilization), polymer encapsulation, and zwitterionic component integration have significantly improved their in vivo durability [47]. Similarly, DNA origami nanostructures may cause mild inflammation or degrade rapidly in biological fluids, but protective coatings such as PEGylation, lipid layers, or virus-inspired capsid-like shells greatly enhance their biocompatibility and stability [48]. Combining these stabilized DNA materials with flexible electronic substrates, including stretchable conductors, screen-printed electrodes, and microfluidic sweat pathways, further ensures long-term performance by maintaining skin contact and stable electrical communication. Overall, these materials and engineering strategies enable DNA-based sensors to operate more reliably under real physiological conditions. Additionally, in 2022, researchers developed a novel microneedle (MN) patch based on stimuli-responsive DNA hydrogels for rapid sampling and sensitive detection of microRNA biomarkers in interstitial skin fluid (ISF) [49]. A DNA hydrogel was assembled from six ssDNA strands, two of which were modified with a fluorophore and a quencher, respectively. The hydrogel was then mixed with methacrylated hyaluronic acid (MeHA) and crosslinked within a microneedle mold to obtain MeHA/DNA-MN patches (Figure 4b). The highly hydrophilic MeHA/DNA matrix enables efficient extraction of ISF. Upon encountering the target miRNA, a DNA strand-displacement reaction is triggered, cleaving crosslinking points and restoring fluorescence, thereby enabling in situ amplified detection of miRNA biomarkers.

**Figure 5:**
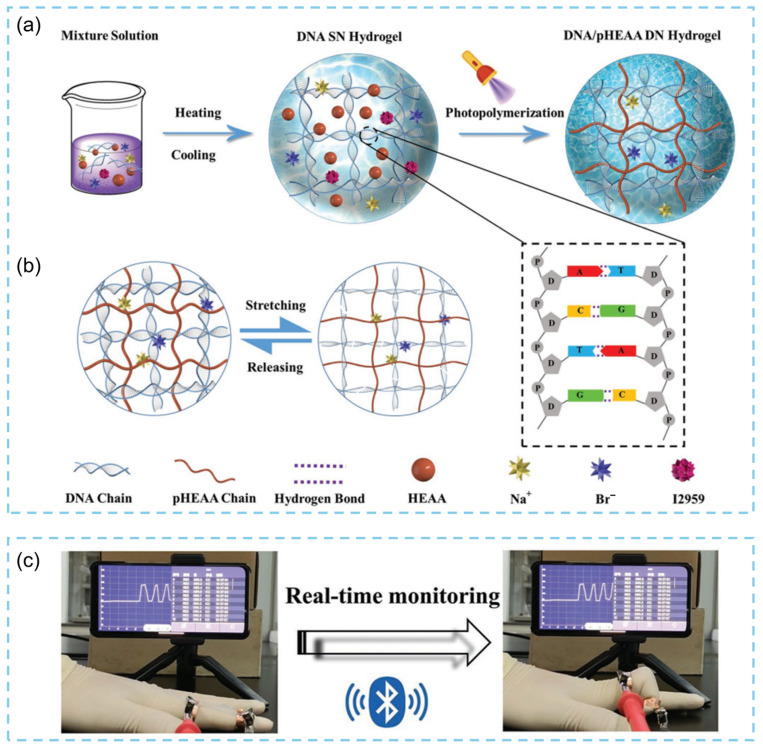
(**a**) Schematic of the DNA/pHEAA DN hydrogel fabrication process. (**b**) Mechanism of the DNA/pHEAA DN hydrogel network structure during stretching and releasing. (**c**) The smartphone receives and detects signals from the wearable electronic skin via Bluetooth [50].

In addition, researchers have explored the use of low-cost, stimuli-responsive DNA hydrogels for sensing applications under extreme environmental conditions. For example, a fully physically crosslinked DNA/p(N-hydroxyethylacrylamide) (DNA/pHEAA) double-network (DN) hydrogel was fabricated using a simple one-pot heating–cooling–photopolymerization process (Figure 5a) [50]. This hydrogel exhibits excellent mechanical properties (maximum tensile stress/strain of 0.96 ± 0.043 MPa/2537.55 ± 23.24%), rapid self-healing capability, outstanding fatigue resistance, and reversible adhesiveness (Figure 5b). When further processed into a wearable electronic skin, it enables real-time monitoring of human motion when integrated with a smartphone (Figure 5c). The electronic skin displays high sensitivity and maintains stable signal output over an ultrawide strain range (0–900%) and during prolonged, high-intensity loading–unloading cycles (500 cycles), demonstrating the strong potential of DNA hydrogels for flexible e-skin applications. In 2020, Liu and colleagues developed another DNA hydrogel based on uracil–adenine nucleobase pairing, which possesses high mechanical robustness, exceptional adhesive properties, and resistance to swelling in both oil and aqueous media [51]. Remarkably, this frost-resistant hydrogel retains excellent flexibility, stretchability, and strong adhesion across a broad temperature range from −20 to 80 °C. Incorporating conductive ions into the hydrogel enables the fabrication of high-performance strain sensors capable of monitoring diverse human motions. Owing to its strong adhesion to moist, oily, or perspiring skin, this DNA hydrogel–based strain sensor provides stable and accurate motion detection even during continuous outdoor activities.

### 2.4. Performance Comparison of DNA-Based Wearable and Implantable Biosensors 

Continuous monitoring with wearable and implantable biosensors demands quick response times, high specificity, low detection limits, and stable operation over long periods in variable physiological or environmental conditions. While DNA hydrogels and DNA origami each bring unique benefits—hydrogels offering soft, tissue-compatible interfaces and origami providing nanoscale spatial control—their effectiveness varies depending on the application. To facilitate quick comparison of existing methods, we summarize key metrics from recent research in Table 1, such as limit of detection (LOD), response time, specificity against biological interferents, and stability during in vivo or ex vivo use. These data underscore the complementary roles of mechanically adaptable DNA hydrogels in sweat and interstitial fluid (ISF) biosensing, and structurally programmable DNA origami for highly sensitive nucleic acid and protein detection.

Overall, the comparison shows that DNA hydrogels are ideal for applications needing extensive signal transduction, flexibility, and biofluid absorption, making them suitable for wearable sweat or ISF sampling. Conversely, DNA origami platforms deliver ultralow LODs and high molecular specificity by precisely arranging probes at the nanoscale and utilizing optical or electrochemical enhancement. The distinct advantages of each system highlight the importance of choosing the right DNA architecture based on target analyte constraints, sensitivity requirements, and operational conditions.

## 3. DNA Origami Nanotechnology

DNA origami technology was first introduced by P. Rothemund in 2006 [55]. Its core concept relies on a bottom-up assembly strategy that folds DNA molecules into programmable nanostructures ranging from several tens of nanometers to the submicrometer scale. In its initial implementation, a long single-stranded DNA from the M13 bacteriophage genome was employed as the scaffold, while hundreds of short “staple strands” were designed to hybridize with specific regions of the scaffold [56,57]. The cross-linking of these staples guided the scaffold to fold into the desired two-dimensional shapes, such as squares, triangles, and stars. The final geometric configuration was entirely determined by the programmed sequence of the scaffold. This high level of programmability not only facilitated the development of computer-aided design tools but also established DNA origami as a versatile, user-friendly, and easily automated nanofabrication technique. As the field advanced, DNA origami evolved from simple 2D constructs to 3D architectures featuring asymmetry, internal cavities, and tunable curvature, enabling the precise fabrication of structures in virtually any desired shape [58]. These nanoscale assemblies exhibit excellent dimensional control, sequence programmability, and biocompatibility, resulting in widespread applications across nanotechnology and biomedicine, including molecular surface patterning [59,60,61], nanoparticle positioning [62,63,64], nanorobotics [65,66,67], targeted drug delivery [68,69,70], molecular recognition [71,72,73], and biosensing. Benefiting from the intrinsic affinity of DNA origami for other nucleic acids and its highly controllable spatial framework, this technique has emerged as a powerful platform for biosensor construction. By extending or functionalizing single-stranded DNA (ssDNA) at predefined positions on the origami scaffold, target molecules, such as biological receptors, signaling probes, or functional materials designed to enhance sensor sensitivity, can be precisely anchored at designated sites. Through this structural programmability and nanoscale spatial precision, DNA origami enables the highly ordered arrangement of probes or functional entities, thereby facilitating the development of next-generation biosensors with improved sensitivity, selectivity, and structural controllability.

### 3.1. Two-Dimensional DNA Origami

#### 3.1.1. Two-Dimensional DNA Origami Design, Synthesis

In the early development of 2D DNA origami, the M13mp18 ssDNA was commonly used as the scaffold. By hybridizing this scaffold with hundreds of carefully designed staple strands, a variety of 2D origami structures could be assembled. However, the limited length of M13mp18 (7249 nt) inherently restricted the achievable size, structural complexity, and geometric diversity of DNA origami constructs [74]. To overcome this constraint, researchers later introduced longer double-stranded DNA (dsDNA), typically obtained through PCR amplification, or long single-stranded DNA as alternative scaffolds [75,76]. These strategies incorporate new scaffold sources and extend scaffold length, significantly expanding the overall dimensions of 2D DNA origami structures. With further technological advances, wireframe 2D DNA origami emerged as a powerful approach. This method employs DNA double helices as “edges” and junctions as “nodes” to assemble open mesh-like frameworks, enabling nanometer-scale precision in the spatial positioning of secondary components and allowing controlled orientation of individual helical segments. Compared with traditional rectilinear brick-like origami, wireframe architectures can form complex polyhedral geometries that are unattainable using conventional duplex-based folding [77,78]. In addition, their open network design greatly reduces the amount of DNA required to construct objects of a given lateral dimension, thereby lowering the dependence on scaffold length. Most importantly, the ability to precisely control helical orientation is crucial for specific applications, such as arranging chromophores to regulate molecular excitonic [79] and photonic behaviors [80].

**Figure 6:**
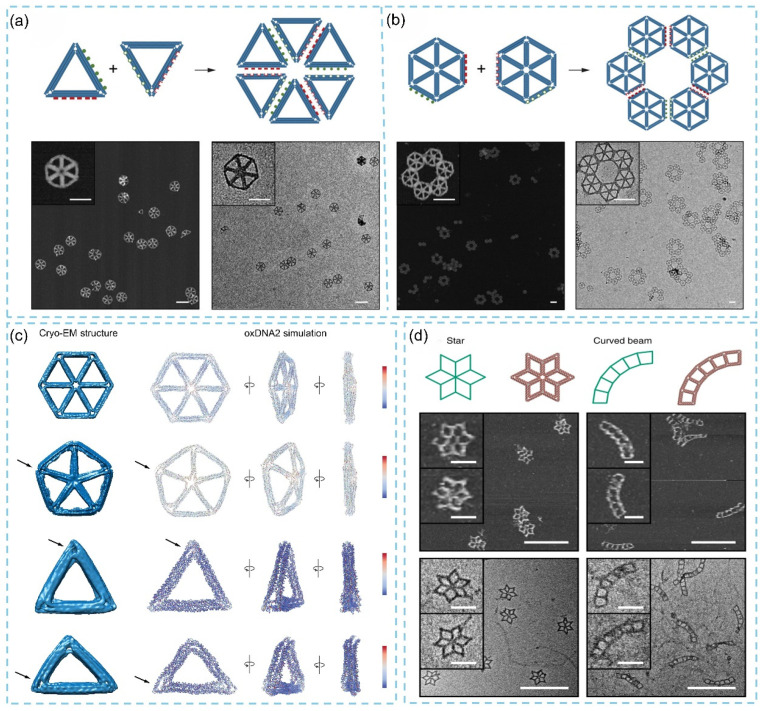
The superstructures of METIS DNA origami formed by (**a**) triangular origami and (**b**) hexagonal origami, scale bars: 100 nm [83]. (**c**) The oxDNA2 coarse-grained simulations and cryo-EM structures for 2D wireframe DNA origami [77]. (**d**) Fully automatic sequence design of scaffolded DNA origami with a star lattice and curved beam lattice from 6HB edges [84].

METIS is a fully automated sequence-design strategy developed for wireframe DNA origami based on six-helix bundle (6HB) edges [81,82]. It constructs lattice-based DNA assemblies using a three-layer architectural scheme that corresponds to the cross-sectional organization of 6HBs and employs a three-way vertex-crossover motif. In this design, each double helix within a given layer is connected to the corresponding helices in adjacent wireframe edges, thereby forming a complete DNA origami structure. In 2022, Wang and colleagues integrated lateral cohesive interactions with a parallel half-crossover strategy between neighboring origami units [83]. Using METIS, they designed a triangular origami structure with 126 bp edges lacking an internal mesh, as well as a hexagonal origami module with 84 bp edges featuring an internal mesh. In both cases, programmed self-assembly successfully yielded size-limited finite superstructures and periodic arrays (Figure 6a,b), demonstrating the versatility of this approach for constructing 2D supramolecular assemblies. In 2022, researchers used single-particle cryo-electron microscopy (cryo-EM) to systematically examine the planarity of 2D wireframe DNA origami designed using the METIS algorithm [77]. The results revealed that regular geometries, such as hexagons and pentagons, exhibited excellent planarity regardless of whether internal mesh reinforcement was used, with lateral dimensions reaching approximately 80 nm (Figure 6c). The researchers further reconstructed an irregular asymmetric triangular structure at a resolution of 13 Å, complementing a previously obtained symmetric triangular model at 10 Å resolution, which is the highest resolution reported to date for 6HB-based DNA origami solved by cryo-EM. These planar 2D DNA origami structures provide highly customizable platforms to incorporate with functional components, such as proteins, chromophores, and nanoparticles, thereby opening exciting opportunities for the development of high-performance biosensors and other advanced nanodevices. Additionally, Jun’s group also employed the METIS automated design platform to generate a variety of 2D DNA origami structures featuring distinct vertex types, including triangular and quadrilateral mesh geometries, designs with or without internal mesh reinforcement, and objects with more complex shapes and topologies [84]. The designed sequences and atomistic models were validated by AFM and TEM imaging, which confirmed the high structural fidelity of the resulting assemblies (Figure 6d). These DNA origami constructs serve as excellent functional platforms for the precise spatial positioning of bioactive molecules, nanoparticles, and proteins at predefined sites, thereby enabling a wide range of biosensor applications.

#### 3.1.2. Two-Dimensional DNA Origami-Based Biosensor Strategies and Effect Mechanism

**Figure 7:**
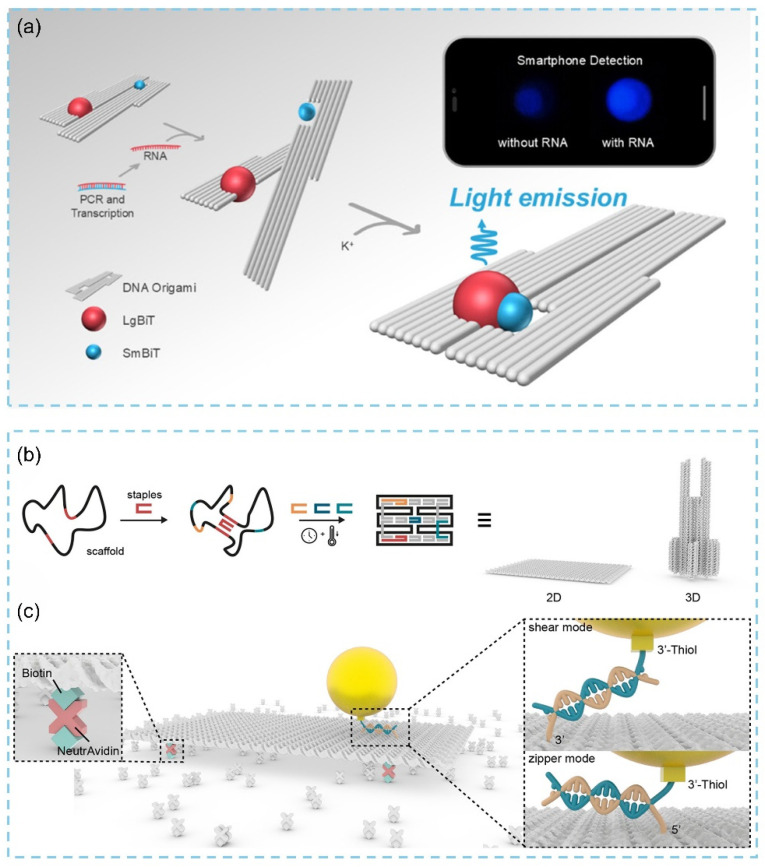
(**a**) Schematic overview of DNA origami-based luminescent biosensors enabling smartphone detection of nucleic acids [52]. (**b**) DNA origami technology to prepare 2D and 3D DNA nanostructures. (**c**) Schematic diagram of 2D DNA origami nanoantenna structure for fluorescence enhancement [85].

In 2025, Kido and colleagues developed a nucleic acid nanosensor based on 2D DNA origami [52]. Upon capturing a target RNA sequence, the device undergoes a conformational change, which is detected using a pair of split luciferase fragments that generate a luminescent signal (Figure 7a). This work introduces a novel biosensing strategy employing dynamic DNA origami devices, offering a promising approach for advancing biosensor technologies. In 2021, Glembockyte’s team addressed the challenge of precisely positioning fluorophores at the nanoscale in biosensors, an issue that often leads to fluorescence quenching, by employing DNA origami to anchor specific fluorophores onto short DNA strands. This enabled the fluorophores to be positioned with nanometer precision on the DNA origami scaffold [85]. Their bottom-up assembly strategy allows for the parallel production of billions of identical nanostructures (Figure 7b). When applying 2D DNA origami nanostructures for single-molecule fluorescence measurements, the researchers further utilized thiol–gold/silver (Au/Ag) interactions to attach ssDNA-functionalized nanoparticles to the origami through zipper-like and shear-like binding modes, thereby achieving tunable distances between the DNA origami and nanoparticles (Figure 7c). Overall, these studies highlight the unique advantages of DNA origami in constructing nanoscale precision scaffolds and complex two-dimensional geometries required for biosensor development, underscoring its potential as a powerful tool for creating high-performance biosensing platforms.

### 3.2. Three-Dimensional DNA Origami

#### 3.2.1. Three-Dimensional DNA Origami Design, Synthesis

**Figure 8:**
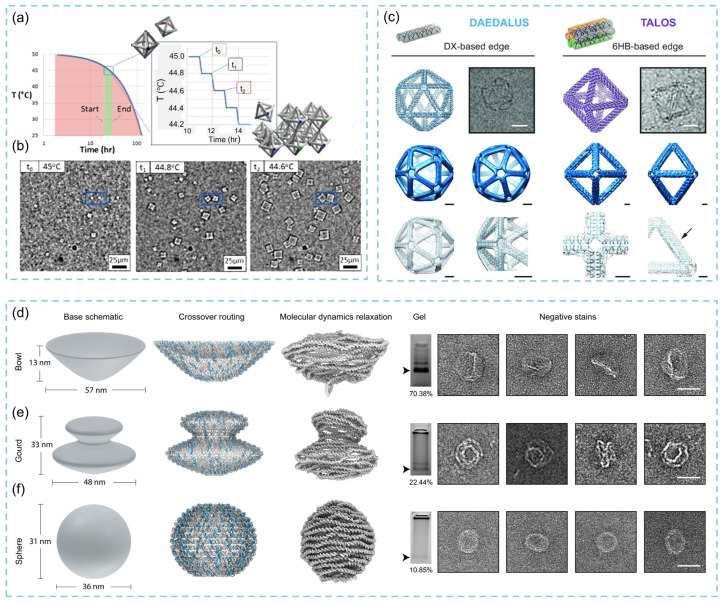
(**a**) Thermal assembly pathway for DNA origami frames from 50 °C to room temperature. (**b**) DNA origami self-assembly into superlattices along the thermal annealing pathway [86]. (**c**) Fully automated sequence design of 3D DNA wireframe structures with DX- and 6HB-based edge [87]. (**d**) Bowl structure design of 3D DNA origami, scale bar, 40 nm. (**e**) Gourd structure design of 3D DNA origami, scale bar, 40 nm. (**f**) Two-layer sphere structure design of 3D DNA origami, scale bar, 40 nm [88].

In 1991, Seeman and colleagues constructed the first 3D DNA nanostructure. Since then, the shapes and design strategies of 3D DNA origami have grown increasingly diverse, and their ability to form large-scale ordered assemblies has opened new possibilities for fabricating complex 3D nanomaterials. Typically, DNA origami relies on Watson–Crick base pairing between complementary single-stranded overhangs to self-assemble into well-defined 3D frameworks under specific conditions. However, balancing attractive interactions during assembly to avoid kinetic traps remains a central challenge in designing efficient assembly systems. Highly ordered DNA nanocrystals are generally crystallized through thermal annealing pathways, and the assembly process is strongly governed by its thermodynamic trajectory. Consequently, thermal annealing is crucial for improving the structural order of assembled frameworks. Traditional annealing protocols often require several days, and such prolonged timescales not only reduce assembly efficiency but also hinder investigations into crystallization pathways, highlighting the need to better understand the factors that influence annealing success. In 2025, researchers combined optical microscopy with small-angle X-ray scattering (SAXS) to systematically analyze the effects of thermal annealing profiles on assembly behavior [86]. Time-resolved optical microscopy revealed minimal crystal evolution within the red region of the annealing curve, whereas active nucleation and growth occurred in the green region, which contains the complete annealing pathway (Figure 8a). Beginning from the initial nucleation event appearing at time t_0_, continuous crystal growth was observed, followed by additional nucleation at t_1_, with both growth and nucleation ceasing at t_2_ (Figure 8b). These observations demonstrate that the assembly of 3D DNA origami frameworks can be described by classical nucleation–growth theory, providing a theoretical basis for guiding crystal formation. Researchers also used the open-source (GNU GPLv3) GUI software of ATHENA to enable fully automated sequence design of arbitrary wireframe 2D and 3D DNA origami structures, supporting both double-crossover (DX) and six-helix bundle (6HB) edge architectures with customizable edge lengths and vertex angles [87]. The 6HB pentagonal design was validated through AFM, TEM, and coarse-grained oxDNA simulations, while the DX-based asymmetric octahedron design was confirmed via cryo-EM (Figure 8c). Traditional 3D DNA origami typically treats helical bundles as straight components aligned along a common vector. To overcome this limitation, in 2025, researchers introduced curved-helix strategies for constructing 3D origami, enabling the creation of capsule-like closed structures [88]. Incorporating curvature not only allows finer discretization of addressable sites and geometric features but also facilitates the construction of larger enclosed volumes that are more suitable for hosting bioactive molecules, producing shapes closer to natural or globular forms. However, the high design complexity associated with curved architectures has restricted their broader application. To address this challenge, a computer-aided design (CAD) tool named DNAxiS was developed to automate the design of axisymmetric DNA nanostructures in 2025. This automated workflow significantly simplifies previously cumbersome design steps, expands the accessible design space for closed curved DNA structures, and resolves earlier limitations related to shape selection, assembly yield, mechanical rigidity, and structural accessibility. Building on this foundation, researchers proposed an “augmented” multilayer design strategy and designed, analyzed, and experimentally validated a series of 3D curved-surface nanostructures (Figure 8d–f). The results demonstrate that applying multilayer principles only to specific segments of the structure markedly enhances overall yield and stability, providing a pathway toward constructing increasingly complex and precise 3D DNA origami nanomaterials.

#### 3.2.2. Three-Dimensional DNA Origami-Based Biosensor Strategies and Effect Mechanism

**Figure 9:**
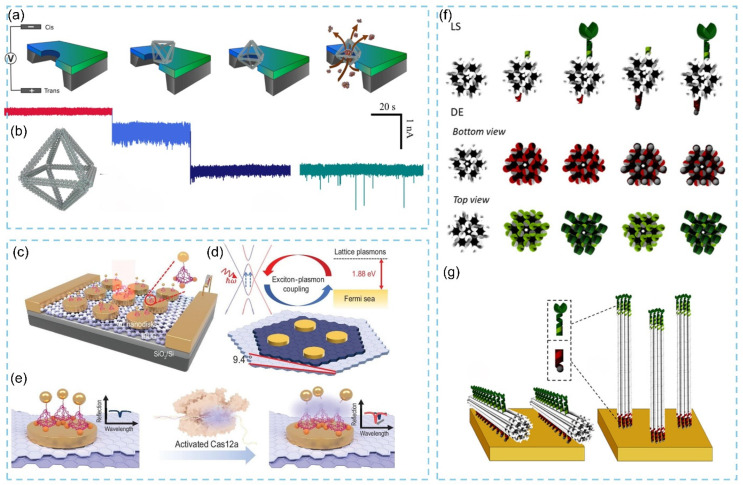
(**a**) Schematic of DNA origami trapping and holo-hSTf translocation through origami trapped hybrid solid-state nanopore. (**b**) 3D Wireframe DNA origami structure [89]. (**c**) Schematic diagram of the structure construction of the optoelectronic biosensor. (**d**) Illustration of the principle of exciton−plasmon coupling. (**e**) Illustration of the principle for miRNA-21 detection [90]. (**f**) 3D DNA origami structures. (**g**) Schematic diagrams of bioreceptor patterning onto a gold surface using DNA origami technology [91].

In 2024, Joly and colleagues introduced a strategy that employs octahedral DNA origami structures to enhance the protein-sensing sensitivity of solid-state nanopore (SSN) biosensors [89]. Using the DNA origami design software caDNAno (http://cadnano.org/, accessed on 18 November 2025), they first constructed 3D octahedral DNA origami (Figure 9a) and then captured the predesigned structures within solid-state nanopores via electrophoresis, thereby generating DNA origami-functionalized SSN biosensors (Figure 9b). By comparing the translocation behaviors of target proteins through open nanopores and hybrid nanopores functionalized with DNA origami, the team performed quantitative analysis of the sensing signals. The results demonstrated that the incorporation of DNA origami significantly increased the capture rate and dwell time of single protein molecules. In 2025, Du et al. [90] developed a hybrid optoelectronic biosensor that uses 3D DNA origami to help integrate gold nanodisk/twisted bilayer graphene (tBLG) heterostructure arrays with the clustered regularly interspaced short palindromic repeat (CRISPR)–Cas12a gene-editing system (Figure 9c). Unlike conventional purely optical detection approaches, this platform exploits exciton–plasmon coupling at the gold nanodisk/tBLG interface to efficiently convert optical signals into electrical responses, achieving a 7 times enhancement in photocurrent compared with pristine tBLG and enabling ultrasensitive detection under low-light conditions (Figure 9d,e). Daems and co-workers further utilized 3D DNA origami to nanopattern the biosensing surface of surface plasmon resonance (SPR) devices, developing a functional thrombin biosensor [91]. They constructed 3D DNA origami structures of varying rigidity (Figure 9f) and functionalized either their distal ends (DE) or lateral sides (LS) with two sets of single-stranded DNA (ssDNA): ssDNA group 1 (shown in red) to specifically anchor the origami structures onto the sensor surface, and ssDNA group 2 (shown in green) to attach thrombin receptors to the origami (Figure 9g). Experimental results showed that both DE and LS configurations maintained excellent stability across defined temperature and pH ranges and enabled nanoscale-precision control over receptor attachment and orientation, leading to substantial enhancement in SPR biosensor performance.

### 3.3. Applications of DNA Origami in Biosensors

#### 3.3.1. Protein Detection

**Figure 10:**
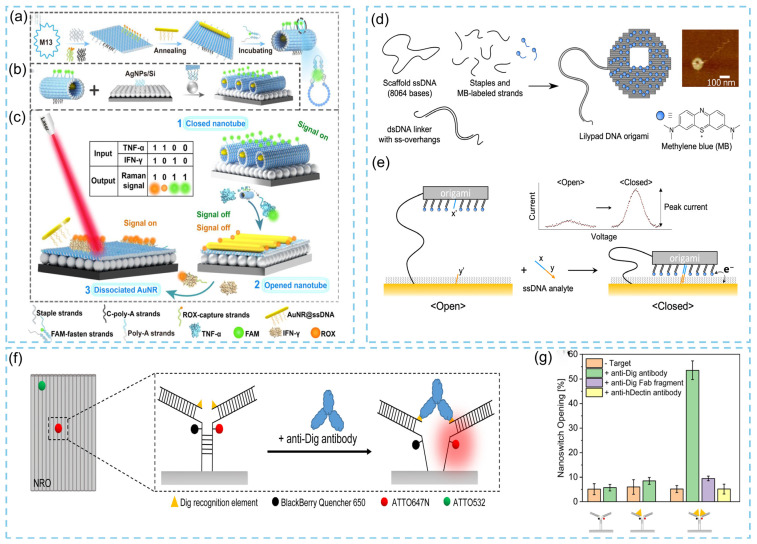
(**a**) The assembly of DNA origami plasmonic nanotubes. (**b**) Combination of DNA origami plasmonic nanotubes with silver nanoparticle-modified silicon wafers (AgNP/Si). (**c**) Schematic illustration of the working principle of the constructed logic gates [92]. (**d**) The design and fabrication method of a Lilypad DNA origami. (**e**) Two types of thiol-modified ssDNA are immobilized on a template-stripped gold surface [93]. (**f**) Schematic representation of the 2D rectangular DNA origami (NRO) nanostructure with an incorporated nanoswitch. (**g**) The signal strength was enhanced around 60 times in the hotspot region of the DNA nanoantenna [94].

DNA origami-assembled plasmonic nanostructures can position target analytes precisely at plasmonic hotspots through sequence-programmable binding sites, thereby enabling highly sensitive molecular recognition. In 2024, a study reported the design and construction of a novel stimulus-responsive DNA origami plasmonic nanoantenna capable of simultaneously monitoring multiple cytokines for cancer immunotherapy applications [92]. This nanoantenna is built upon a hollow DNA origami plasmonic nanotube, which is formed by fastening gold nanorods (AuNRs) along the long edges of a DNA origami nanosheet (Figure 10a,b). Furthermore, the authors implemented a complete Boolean logic gate system that uses cytokine concentrations as input signals and outputs corresponding Raman spectral changes (Figure 10c). The measured levels of TNF-α and IFN-γ in sera collected from tumor-bearing mice were consistent with those obtained using conventional enzyme-linked immunosorbent assays (ELISA). This strategy is therefore expected to accelerate the development of DNA origami-based biosensors for the detection of diverse biomolecules. In 2025, Jeon et al. [93] integrated the advantages of reconfigurable DNA origami and aptamer-based electrochemical aptamer-based (E-AB) sensing systems to develop a one-step, reagent-free electrochemical biosensing platform entirely constructed from DNA origami (Figure 10d). By simply replacing the analyte-binding domain, the platform can be readily adapted to detect a broad range of molecular targets. The researchers combined a 2D planar DNA origami structure with a double-stranded DNA (dsDNA) linker to create a “water-lily” configuration. In this system, DNA origami decorated with multiple redox reporters of methylene blue (MB) is tethered to an ultraflat gold electrode through a long and flexible dsDNA linker. Upon analyte binding, the initially open conformation switches to a closed state by bridging the origami and electrode-bound probe. This conformational transition can be monitored by square-wave voltammetry (SWV). Because SWV current depends on the electron-transfer rate between MB molecules and the electrode and varies strongly with distance, the water-lily architecture enables direct, single-step quantification of nucleic acids and proteins (Figure 10e). In 2021, researchers developed a single-molecule antibody sensor by integrating a nanoswitch recognition module into a DNA origami nanostructure [94]. A simple 2D rectangular DNA origami (NRO) served as the platform, providing highly accessible sites for constructing antibody-recognition units. During the folding process, the authors embedded a nanoswitch into the NRO structure. The nanoswitch consists of two ssDNA strands extending from the origami, followed by a 5-nucleotide complementary sequence that forms a short stem. Each strand is modified with ATTO 647N or BlackBerry Quencher 650 (BBQ-650), forming a fluorophore–quencher pair, and terminates in a Dig-labeled ssDNA anchoring domain, enabling recognition of anti-digoxigenin (Dig) antibodies (Figure 10f). Experiments demonstrated that this sensor can specifically detect sub-nanomolar levels of anti-Dig antibodies within minutes. When the nanoswitch was integrated into the hotspot region of a DNA nanoantenna, the signal intensity was enhanced by approximately 60 times (Figure 10g). This work significantly improved the detection limit of single-molecule protein sensing platforms and demonstrated strong potential for integration with signal-amplification strategies.

#### 3.3.2. Nucleic Acid Detection

**Figure 11:**
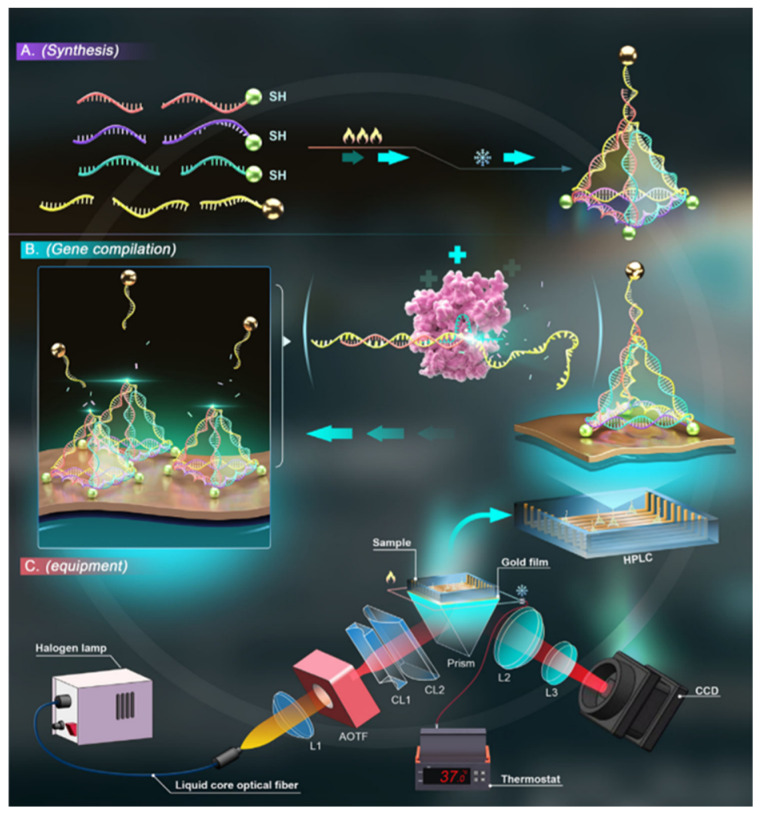
Schematic diagrams of the design of SPR biosensing incorporating DNA origami and DNA scissors [53].

In 2024, Chen and colleagues reported a significant advancement by integrating CRISPR (clustered regularly interspaced short palindromic repeats) technology with DNA origami and coupling it efficiently with surface plasmon resonance (SPR) sensing [53]. CRISPR provides single-nucleotide resolution for sequence recognition, while SPR offers exceptional detection sensitivity; the synergistic combination of these two techniques greatly enhances molecular diagnostic performance. Meanwhile, the DNA origami–based probe architecture overcomes the inherently low trans-cleavage efficiency of CRISPR on solid surfaces, thereby ensuring robust system operation. This hybrid platform enables the detection of ultralow-abundance circulating tumor DNA (ctDNA) and allows precise discrimination of point mutations within gene sequences, demonstrating the feasibility and advantages of CRISPR–SPR integrated sensing. As illustrated in Figure 11, during the synthesis stage, three sets of thiol-modified ssDNA strands are immobilized onto a gold-coated sensing surface via strong Au–S interactions, while a fourth ssDNA strand containing a poly(A) sequence binds to gold nanoparticles (AuNPs) (Figure 11A). In the gene compilation stage (Figure 11B), the presence of target DNA activates Cas-mediated cleavage of ssDNA components within the DNA origami probe, releasing the encapsulated AuNPs. Detachment of AuNPs from the surface reduces the local surface charge density, leading to a corresponding decrease in the SPR signal. This change can be monitored in real time using a high-speed spectroscopic SPR system (Figure 11C), enabling highly sensitive detection of ctDNA at ultralow concentrations.

**Figure 12:**
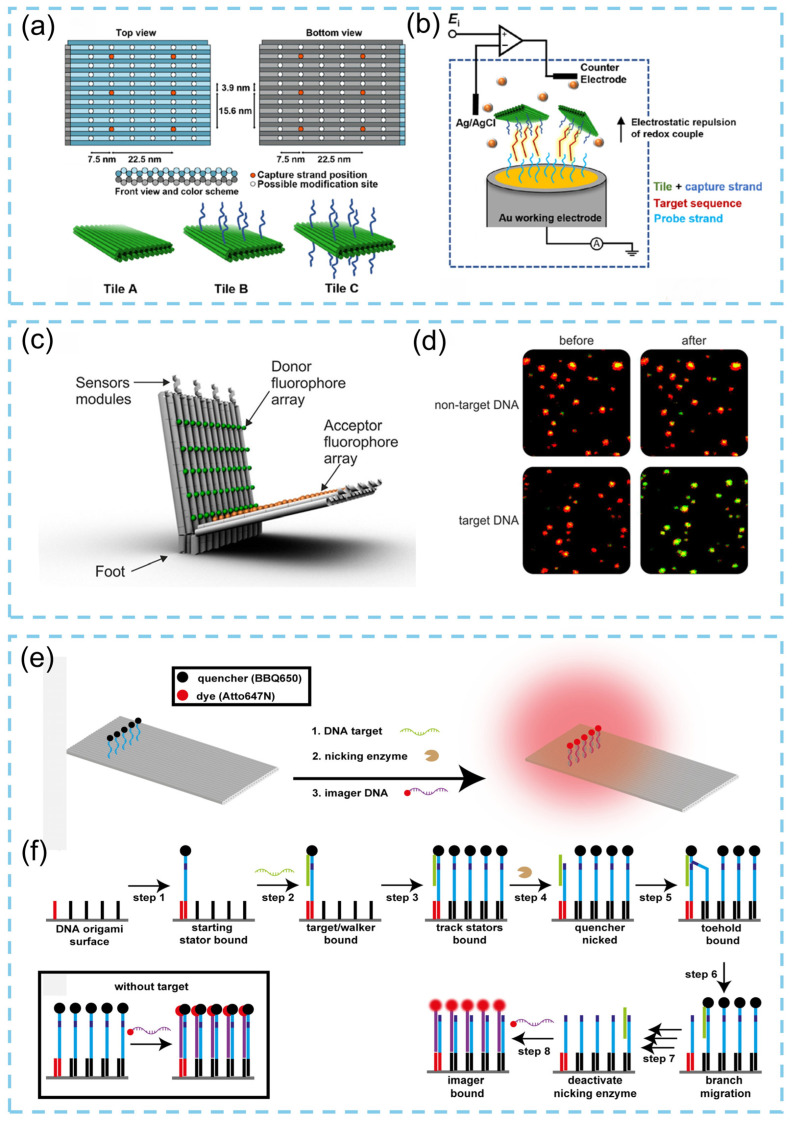
(**a**) Design of the DNA origami structure and used in signal amplification in biosensors. (**b**) Schematic diagram illustrating DNA origami structures that amplify signals from biosensors [95]. (**c**) Design of the DNA origami multifluorophore beacon. (**d**) Fluorescence images recorded before and 2 min after addition of nontarget or target DNA [96]. (**e**) Schematic illustration of the DNA walker on the DNA origami. (**f**) The fluorescence amplification mechanism and action mechanism of DNA walkers [97].

In 2023, a study reported a programmable DNA origami chip that markedly enhances the sensitivity of DNA detection [95]. This pinboard-like DNA origami chip, designed using caDNAno, serves as a simple and highly modular platform (Figure 12a) that can be interfaced with functionalized electrodes (FE), composed of surface-immobilized probes and polycrystalline gold electrodes (PGE) (Figure 12b), depending on the target analyte. When integrated into conventional Faradaic, label-free electrochemical biosensing strategies, these nanostructured assemblies significantly improve detection performance, extending the linear working range of the sensor into the low picomolar (pM) regime while maintaining excellent selectivity. More importantly, coupling this sensing architecture with low-cost, disposable thin-film gold electrodes provides a promising foundation for scalable fabrication and broad practical implementation. Selnihhin and colleagues [96] reported the design, assembly, and characterization of a DNA origami–based optical biosensor incorporating precisely arranged arrays of fluorescent dyes for quantitative DNA detection. In this system, donor and acceptor fluorophore arrays form multiple fluorophore-based Förster resonance energy transfer (FRET) pairs, generating intense signal outputs suitable for single-device microscopic analysis. The incorporation of localized, cascade-type hybridization chain reactions (HCR) on the DNA origami platform further enhanced sensor performance, and this cascading module could be readily integrated into the sensing units of DNA origami beacons. The resulting DNA origami beacon produced amplified fluorescence signals, enabling the direct visualization of individual devices using standard fluorescence microscopy. The core concept involves constructing regularly spaced donor–acceptor fluorophore arrays within the DNA origami structure to generate multichromophoric FRET pairs and thereby amplify fluorescence output. As shown in Figure 12c, the DNA origami beacon, designed using caDNAno, consists of two rectangular panels: a bottom panel anchored by “feet” and a top panel connected via multiple sensing modules. Precise placement of fluorophores is achieved through the DNA origami architecture, and the donor–acceptor arrays collectively generate strong microscopic detection signals. Experimental measurements revealed that the average FRET value remained unchanged upon the addition of non-target DNA, whereas target DNA induced a marked increase in FRET efficiency, which was directly observable in fluorescence images (Figure 12d). Using this strategy, quantitative detection of target DNA at concentrations as low as 100 pM can be achieved within 30 min. In addition, Tinnefeld’s group [97] constructed a 2D rectangular DNA origami structure (approximately 100 nm × 70 nm) using an M13mp18-derived scaffold and 192 staple strands, and employed it as a track platform for a DNA “walker.” DNA sequences tethered to the origami track served as the “walker” units. The study revealed that the DNA walker functions as a linear fluorescent signal amplifier by catalyzing enzymatic nicking reactions for highly sensitive nucleic acid detection. As illustrated in Figure 12e, in the presence of target DNA, the walker migrates along the track with the assistance of a nicking enzyme, cleaving quencher-labeled stator strands. Visualization is enabled by an Atto647N-labeled imaging strand that hybridizes with the stator; fluorescence is emitted only when the quencher is removed during the walking process. In the absence of target DNA, the imaging strand remains in proximity to the quencher, preventing fluorescence emission (Figure 12f). The resulting fluorescence intensity distribution on the DNA origami allows discrimination between walker sequences differing by a single nucleotide, demonstrating the system’s high sequence specificity.

#### 3.3.3. Virus Detection

**Figure 13:**
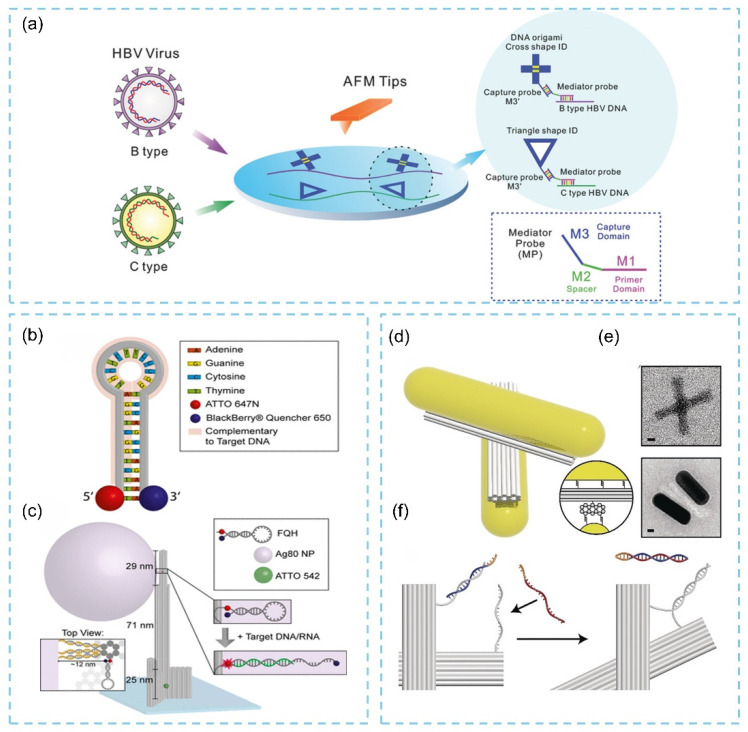
(**a**) Schematic illustration of AFM-based HBV genotyping matching with DNA origami shape IDs [98]. (**b**) DNA-based fluorescence-quenching hairpin (FQH) is used for the detection of Zika. (**c**) Biotin-modified DNA origami pillar immobilized on a BSA-biotin/neutravidin surface [99]. (**d**) Schematic diagram of the DNA origami structure. (**e**) TEM images of DNA origami. (**f**) Schematic diagram of viral molecular recognition mechanism [100].

Liu and colleagues combined DNA origami technology with the high-resolution recognition capability of atomic force microscopy (AFM) to develop a nucleic acid detection strategy based on the shape recognition of DNA nanostructures [98]. This method enables the identification of multiple genetic mutations on the hepatitis B virus (HBV) target DNA and allows the precise discrimination of different genotypes at the single-molecule level (Figure 13a). Their findings indicate that the labeling efficiency of HBV is strongly dependent not only on the geometry of the DNA origami structure but also on the number and spatial arrangement of the capture probe M3′. An optimized origami configuration reduces electrostatic repulsion between the nanostructure and the viral target, whereas the spatial placement of the probes influences capture efficiency due to steric hindrance. Increasing the number of probes enhances collision probability, thereby improving overall detection performance. Sensitivity evaluations showed that this method achieves a detection limit as low as 10 × 10^−12^ M (0.34 ng mL^−1^), demonstrating its reliability, high sensitivity, and promising potential for HBV genotyping and viral load quantification. In addition, Ochmann et al. [99] developed a physical fluorescence amplification mechanism based on DNA origami. By immobilizing noble metal nanoparticles on DNA origami scaffolds and allowing a DNA “walker” to traverse the origami nanoantenna, plasmonic hotspots are generated along its path, resulting in substantial fluorescence enhancement (Figure 13b,c). Using this modular strategy, the team successfully detected Zika virus DNA and RNA in human serum. These results highlight the modularity of DNA nanotechnology, which enables the integration of multiple functional components into a “DNA nanolab,” thereby simplifying the detection of low-abundance analytes in point-of-care settings. Meanwhile, Liedl’s group employed a reconfigurable DNA origami template to assemble chiral gold nanorods (AuNRs) for the highly sensitive detection of microRNA and viral RNA at concentrations as low as 100 pM [100]. The sensing unit consists of a 3D gold–DNA hybrid structure formed by two “arms” connected through single-stranded DNA, each arm anchoring an AuNR (Figure 13d,e). Depending on the arm orientation, the entire hybrid adopts either left- or right-handed chirality, producing distinct circular dichroism (CD) signals. Single-stranded oligonucleotides positioned at the arm termini serve as recognition elements. In the absence of target RNA, the structure remains locked in a right-handed configuration; however, upon target binding, a strand-displacement reaction induces a switch to the left-handed state, generating a strong CD response (Figure 13f). This chirality-based nanosensor provides a novel strategy for the sensitive detection of pathogenic RNA without the need for target amplification.

### 3.4. System-Level Integration: Flexible Electronics, Microfluidics, and Wireless Readout

Recent advancements in wearable and implantable biosensors indicate that bringing DNA hydrogels and DNA origami into practical applications requires more than just developing new materials. It also involves combining these materials with flexible electronics, microfluidic devices, and wireless tech [101]. These interdisciplinary approaches enable ongoing sampling, dependable signal transfer, and remote data gathering, essential capabilities for real-time, in situ biomedical monitoring.

*Integration with flexible electronics*. DNA hydrogels are increasingly integrated with flexible electronic substrates, such as elastomer-based stretchable conductors, conductive polymer networks, carbon-based inks, and screen-printed electrodes [102,103]. These materials maintain their electrical conductivity during bending, twisting, or stretching, which are common in skin-mounted devices. Hybrid assemblies of DNA hydrogels with electrodes facilitate both biochemical detection and mechanical flexibility. Encapsulation with silicone elastomers or polyurethane films further improves long-term adhesion and reduces dehydration during extended use [104]. Additionally, DNA origami nanostructures have been attached to ultraflat gold or graphene electrodes through thiol–gold interactions, enabling precise nanoscale placement of capture probes to enhance electrochemical sensitivity.

*Integration with microfluidics*. Microfluidic preprocessing modules offer precise control over sample handling, including delivery, enrichment, and filtration. This is particularly useful for detecting low-level biomarkers in sweat, saliva, and interstitial fluid. DNA hydrogels can be incorporated into microchambers to act as responsive valves or concentration matrices, aiding in the pre-filtration of ions or proteins before detection [105]. In DNA origami systems, microfluidic channels help maintain stable reaction environments and minimize nonspecific interactions, thereby improving the accuracy of CRISPR-based cleavage, DNAzyme activity, and plasmonic signal enhancement. Furthermore, modular microfluidic platforms support multiplexing, allowing the integration of multiple DNA sensors into a single portable diagnostic device.

*Wireless and battery-free readout*. Moving from laboratory setups to practical biosensing solutions requires wireless signal transfer compatible with everyday electronics. Several DNA hydrogel sensors already connect to near-field communication (NFC) resonators [106], enabling battery-free detection via smartphones; changes such as DNA degradation or swelling alter the resonator’s dielectric profile, leading to measurable frequency shifts. Likewise, DNA origami–based electrochemical sensors can continuously communicate with low-energy Bluetooth transmitters, enabling continuous data transmission. Smartphone cameras can also capture optical signals from luminescent or plasmonic DNA origami using simple lens attachments. These systems reduce hardware complexity and support decentralized diagnostics, ideal for home health monitoring or remote clinical supervision.

These integration strategies emphasize the increasing overlap between programmable DNA nanomaterials and system-level engineering. As advances in electronics, microfluidics, and wireless modules occur, DNA hydrogels and origami will increasingly serve as intelligent biochemical interfaces in fully autonomous wearable and implantable biosensing devices.

## 4. The Challenges of DNA Technology

Despite significant advances in the development of stimuli-responsive DNA hydrogels in recent years, their performance and practical applications still require substantial improvement. First, DNA supramolecular hydrogels inherently exhibit low storage modulus, and research on strategies to enhance their mechanical properties remains limited, largely due to methodological constraints. Nevertheless, mechanical robustness is critical for the functional deployment of hydrogels. Current approaches to tuning the mechanical properties of DNA hydrogels primarily rely on strengthening physical interactions and introducing chemical crosslinking. Chemical crosslinking transforms non-covalent interactions into stable covalent bonds, thereby markedly improving mechanical strength. However, hydrogels crosslinked through permanent covalent bonds typically lose injectability and self-healing capability, preventing autonomous shape recovery in vivo and necessitating surgical implantation [107]. These limitations severely hinder their biomedical applications.

While these limitations are well understood, the fundamental mechanistic reasons behind them are often overlooked. In DNA hydrogels, a significant obstacle is the difficulty in precisely controlling cross-linking density at the molecular level. Minor variations in oligonucleotide sequence design, base-pairing thermodynamics, or hybridization kinetics can lead to irregular network formation, resulting in unpredictable mechanical properties and making large-scale reproducibility challenging. In DNA origami systems, synthesizing and purifying long scaffold strands and numerous staple sequences remain significant hurdles, as more extended sequences tend to increase errors, reduce folding efficiency, and complicate staple overhang design for functionalization. Moreover, both DNA hydrogel and origami manufacturing face scaling challenges due to their reliance on slow thermal annealing processes, which require narrow temperature windows for proper hybridization and cause yield losses when scaled up. These molecular and process-level constraints pose fundamental problems that must be addressed to enable dependable production and broader application of DNA-based biosensing platforms.

Wearable and implantable biosensors must endure dynamic physiological and environmental conditions, and DNA-based materials exhibit several deployment-dependent vulnerabilities that must be addressed to enable real-world translation. For wearable platforms, continuous exposure to sweat can accelerate hydrogel dehydration, promote ion-induced structural collapse, and weaken adhesive interfaces, particularly during extended physical activity or repetitive mechanical bending. Long-term skin-mounted devices also undergo significant mechanical abrasion, which can disrupt the integrity of the hydrogel network or alter conductive pathways in hybrid hydrogel–electrode systems. For implantable sensors, enzymatic degradation poses a significant

Endogenous nucleases rapidly cleave the DNA hydrogel network, shortening device lifetime and reducing signal stability. Several studies have therefore introduced nuclease-shielding strategies such as polymer encapsulation, chemical backbone modification (e.g., phosphorothioate substitution), and cationic polymer coatings to improve in vivo durability. DNA origami nanostructures also face immunogenicity concerns, as unmethylated DNA motifs may activate innate immune pathways. However, recent reports demonstrate that techniques such as PEGylation, lipid-layer cloaking, and sequence-level immunogenic site removal markedly reduce macrophage uptake and cytokine activation, allowing more stable blood- or tissue-resident operation. These considerations emphasize that engineering solutions addressing degradation, abrasion resistance, and biocompatibility are essential for the successful deployment of DNA hydrogels and DNA origami in practical biosensing systems. Furthermore, recent studies highlight that assessing biosensor performance involves more than just attaining very low detection limits; factors such as practical reliability, noise resistance, and accurate sensing within context are equally important [108].

Furthermore, such hydrogels are difficult to repair once damaged by external forces, increasing operational risks and shortening service life. In addition, the environmental stability of stimuli-responsive DNA hydrogels remains a major challenge. Specifically, their poor tolerance to harsh conditions, such as low temperatures [109,110] or organic solvents [111], greatly restricts their applicability in certain environments. Finally, the high cost and limited synthetic efficiency of DNA hydrogels currently impede large-scale production. DNA origami technology offers exceptional innovation, versatility, and precision for biosensing applications, yet its scalability and practical use are hampered by several barriers. The fabrication cost is extremely high, due to the expensive price of long DNA scaffolds, and the yield of the resulting nanostructures is relatively low. Each application typically demands customized DNA origami designs, consuming substantial time and resources. The dependence on advanced characterization techniques, such as atomic force microscopy, cryogenic electron microscopy, and fluorescence imaging, further increases cost and operational complexity, limiting routine use. Additionally, DNA origami structures are highly sensitive to environmental conditions and display poor stability under fluctuating temperatures or extreme chemical environments (e.g., strong acids or bases), remaining intact only under mild conditions. Lastly, the limited diversity of available DNA scaffolds constrains the dimensionality and complexity of DNA origami structures. The mechanisms governing the growth of polymers or inorganic materials directed by DNA origami templates also remain unclear, posing additional challenges to the further development of this technology.

## 5. Conclusions and Outlook

From soft, deformable adaptive hydrogels to precisely programmable DNA origami nanostructures, the integration of DNA materials with biosensing technologies provides a reliable pathway for continuous and efficient molecular monitoring in practical applications. Recent research has primarily focused on two directions: (1) Integration of DNA hydrogels into wearable biosensors. Both pure DNA hydrogels and hybrid hydrogels incorporating additional materials exhibit excellent stimuli-responsive behavior. They undergo structural or functional changes upon exposure to specific molecular cues, enabling highly sensitive detection of target analytes. These properties make DNA hydrogels particularly suitable for wearable sensing platforms aimed at continuous monitoring of biomarkers in sweat or other body fluids. (2) DNA origami for highly flexible and precise biosensing. DNA origami enables the programmed self-assembly of DNA into well-defined 2D or 3D nanostructures, providing highly controllable molecular platforms. The structural diversity of DNA origami supports the design of various types of biosensors capable of accurate detection of proteins, nucleic acids, viruses, and other biomolecules. Advancing DNA-based wearable and implantable biosensors depends on more focused, technology-driven design approaches. AI tools for modeling and sequence optimization can make constructing durable DNA hydrogels and accurately folding DNA origami structures more efficient, while synthetic biology enables the creation of nuclease-resistant or chemically modified DNA with better stability inside the body. Practical application needs should direct material choices, such as prioritizing long-term hydration, antifouling, and mechanical resilience for wearable sweat sensors, and emphasizing biocompatibility, immune evasion, and enzymatic resistance for implants. Moreover, digital twin simulations and integrated modeling platforms can accelerate the prediction of device behavior in realistic biological environments. These strategies offer a more straightforward path for converting DNA nanomaterials from conceptual ideas into fully operational biosensors.

Emerging technological advances clearly outline actionable pathways to surpass current limits in DNA-based wearable and implantable biosensors. AI-driven design of DNA nanostructures now facilitates quick predictions of folding routes, mechanical properties, and hybridization energetics, greatly minimizing trial-and-error efforts in creating complex DNA hydrogels and origami structures. Synthetic biology offers promising methods for large-scale production of nuclease-resistant or chemically modified DNA, such as enzymatically synthesized XNA and backbone-modified DNA analogs with improved in vivo stability. Future efforts should focus on application-specific needs: for wearable sweat sensors, long-term hydration, antifouling, and mechanical resilience are essential; for implantable devices, biocompatibility, immune evasion, and enzymatic resistance are paramount. Recent progress in digital twin simulation platforms for biosensors can further accelerate development by enabling virtual testing of fluid flow, signal transmission, and deformation under physiological conditions. These technological directions form a roadmap to transition DNA hydrogels and DNA origami from laboratory prototypes to reliable, clinically deployable biosensor platforms. Given the current challenges, the future development of DNA hydrogels should focus on: (i) establishing efficient and cost-effective methods for DNA synthesis and modification to enable large-scale production and industrial translation; (ii) enhancing the mechanical strength, self-healing capability, and environmental stability of DNA hydrogels to improve their robustness, durability, and suitability for wearable and implantable devices. The future advancement of DNA origami techniques should prioritize: (i) developing user-friendly, automated, and high-throughput design tools and characterization methods to streamline the fabrication process and reduce costs; (ii) expanding the library of DNA scaffolds or exploring alternative construction strategies to create larger and more complex multidimensional structures; (iii) improving the environmental stability of DNA origami to maintain functional integrity under varying temperature, pH, and ionic conditions, thereby enhancing the stability, adaptability, and scalability of DNA origami-based biosensors.

Overall, the combination of DNA hydrogels and DNA origami offers a promising strategy for developing portable, highly specific, and low-cost biosensing platforms. Such sensors hold tremendous potential in fields ranging from medical diagnostics to environmental monitoring. With continued research growth, further breakthroughs are expected in the near future.

## Figures and Tables

**Table 1:** Comparative performance metrics of DNA-based wearable and implantable biosensors.

Sensor Type	Target Analyte	Material Platform	LOD	Response Time	Specificity	Stability (In Vivo/Ex Vivo)	Ref.
Wearable DNA hydrogel-based electrochemical sensor	IFN-γ in sweat	Aptamer–DNA hydrogel on SPCE	0.1–1 pg/mL	<10 min	High; aptamer-specific displacement, minimal cross-reactivity	Stable during 2–4 h of continuous sweat exposure	[46]
DNA hydrogel–microneedle patch	microRNA in ISF	MeHA/DNA hybrid hydrogel	~10 fM	5–15 min	High; strand-displacement discrimination between single-base variants	Maintains mechanical integrity during 24 h skin penetration	[49]
Wearable infection-monitoring DNA gel	Bacterial DNase	DNase-responsive DNA hydrogel	Qualitative (visual RF shift)	Seconds–minutes	High; DNase-triggered degradation only	Stable on skin for >48 h; tolerates mechanical bending	[45]
DNA origami luminescent nanosensor	Viral RNA	Two-dimensional DNA origami + split luciferase	~100 fM	<5 min	Extremely high; sequence-level precision	Stable for hours at physiological ionic strength	[52]
DNA origami SPR biosensor	ctDNA (point mutations)	DNA origami + AuNP SPR interface	1–10 fM	<10 min	Single-nucleotide discrimination	Stable over 12 h continuous flow	[53]
DNA origami chiral plasmonic nanosensor	Viral RNA/miRNA	Reconfigurable AuNR–DNA origami	~100 pM	Seconds	Sequence-specific switching	Stable after repeated optical cycling	[54]

## Data Availability

No new data were created or analyzed in this study.
